# The complex relationships involved in global health: a qualitative description

**DOI:** 10.1186/1472-6920-13-136

**Published:** 2013-10-03

**Authors:** Anne E McCarthy, Andrew Petrosoniak, Lara Varpio

**Affiliations:** 1 Department of Medicine, Faculty of Medicine, University of Ottawa Ottawa Hospital General Campus, 501 Smyth Rd, K1H 8L6 Ottawa, ON, Canada; 2 Office of Global Health, Faculty of Medicine, University of Ottawa, 451 Smyth Road, K1H 8M5 Ottawa, ON, Canada; 3 Emergency Medicine Residency Program, Division of Emergency Medicine, University of Toronto, 2075 Bayview Ave, M4N 3M5 Ontario, Canada; 4 Academy for Innovation of Medical Education, Faculty of Medicine, University of Ottawa, 451 Smyth Rd, K1H 8M5 Ottawa, ON, Canada

**Keywords:** Global health, Trainees, Medical education

## Abstract

**Background:**

Growing numbers of medical trainees now participate in global health experiences (GHEs) during their training. To enhance these experiences we sought to explore expectations inherent in the relationships between GHE stakeholder groups.

**Methods:**

20 open-ended, semi-structured interviews probed participant perceptions and assumptions embedded in GHEs. A fundamental qualitative descriptive approach was applied, with conventional content analysis and constant comparison methods, to identify and refine emerging themes. Thematic structure was finalized when saturation was achieved. Participants all had experience as global health participants (10 trainees, 10 professionals) from an urban, academic, Canadian medical centre.

**Results:**

We identified three stakeholder groups: participants (trainees and professionals), host communities, and sponsoring institutions. During interviews, four major themes emerged: (i) cultural challenges, (ii) expectations and perceptions, (iii) relationships and communication, and (iv) discordant objectives. Within each theme, participants recurrently described tensions existing between the three stakeholder groups.

**Conclusions:**

GHE participants frequently face substantial tensions with host communities and sponsoring agencies. Trainees are particularly vulnerable as they lack experience to navigate these tensions. In the design of GHEs, the needs of each group must be considered to ensure that benefits outweigh potential harms. We propose a conceptual model for developing educational objectives that acknowledge all three GHE stakeholder groups.

## Background

Global health is increasingly recognized as a core element of medical education [1] with greater numbers of under- and post-graduate medical trainees taking part in international global health experiences (GHEs) [2-6]. Recent surveys determined that all Canadian medical schools permit undergraduate medical students to participate international GHEs and the majority (94%) provide some access to pre-departure training. However, 44% of schools allow students to arrange international electives without faculty support or supervision [7-9]. This is rather alarming. Although medical educators increasingly acknowledge the benefits of GHE participation [3,4,6,9], there are potential harms [7,8,10]. Participants may perform skills beyond their training level placing patients at increased risk [11]. Trainees may also suffer threats to their own health and safety [12]. Consequently, targeted training is crucial to adequately prepare trainees for GHEs [13,14]; yet, fewer than 30% of trainees participate in such programs [15,16]. A number of authors have proposed educational strategies [2,13,17] and several groups have developed curriculum guidelines for implementation within medical schools [1,11,14,18]. Some suggest providing “academic, logistic and financial support for international rotations”, and promoting GHEs as a “routine part of medical education” [2]. While there does not yet exist a formal consensus among educators for curriculum content or educational opportunities in global health [19], there is agreement that several topics including context-relevant biomedical competencies [20,21] and cultural sensitivity development [11,22,23] should be included.

This study originated as a needs assessment aimed at exploring our community’s content needs for trainee-oriented GHE pre-departure training. This needs assessment was informed by our study of the global health education literature. We aimed to vet the GHE guidelines currently available by having the GHEs of trainees and medical professionals in our context inform our interpretations.

During data collection for the needs assessment, study participants described content topics for inclusion in our GHE training program. Additionally, and perhaps more importantly, participants continuously emphasized the complex relationships and expectations involved in GHEs that trainees had to navigate. In our literature searches, we found little reported evidence aimed specifically at describing the complicated affiliations and expectations involved in GHEs.

The purpose of reporting of study data is to answer two questions: (1) who are the participant groups involved in GHEs, and (2) what are some of the expectations inherent in the relationships between these groups. Based on these data, we propose a way for conceptualizing the complex relationships involved in GHEs. We suggest that this conceptualization can help GHE developers and participants alike to be better prepared for the unique challenges these learning experiences entail.

## Methods

This study received approval from our institution’s health research ethics board. All participants provided written, informed consent.

### Methodological orientation

We use a fundamental qualitative descriptive approach [24] for this study. Our goal is to present participants’ perceptions of GHE relationships and expectations with minimal transformation. We employed a fundamental qualitative descriptive approach since it draws heavily from naturalistic inquiry, providing techniques for allowing the target phenomenon to present itself in its natural state [24]. It is recognized as particularly amenable to obtaining straight and minimally “spun” answers to questions relevant to practitioners and policy makers [24].

### Setting and participants

This study was conducted at an urban, academic Canadian medical centre. We began recruitment by inviting participants from a convenience sample [25] of individuals known to the researchers for having participated in GHEs. Snowball sampling [25] was used to identify other potential participants. We reasoned that professional status (trainee vs professional) and amount of time spent in GHEs were important criteria that would shape perceptions of the complex affiliations and expectations in GHEs. Consequently, during the snowball sampling phase, purposive sampling [26] with constant comparison of professional status and amount of time spent engaged in GHEs was used to ensure maximum variation. Interviews were conducted until thematic saturation was achieved.

We originally estimated that 30 interviews would be needed to reach thematic saturation. Instead, this was achieved with 20 interviews—10 with medical trainees (5 male; undergraduate = 9; postgraduate =1) and 10 with healthcare professionals (7 male; physicians = 8, occupational therapist = 1, registered nurse = 1). Inclusion criteria required all participants to have had at least one experience working in a healthcare related capacity in a resource-poor setting. Trainees averaged 1.5 GHEs (range: 1–3) while healthcare professionals averaged 8.8 GHEs (range 2–15). The total mean time abroad was 7.6 weeks and 8.1 months for trainees and professionals respectively. The geographical distribution of GHEs was variable with most participants visiting Africa (12 countries) followed by South East Asia (5 countries), Central America (3 countries) and South America (1 country).

### Data collection

A semi-structured, open-ended interview protocol was developed with questions aimed at enabling participants to describe as much of their GHE as they considered relevant. Questions probed the conditions, processes, goals and factors of the GHE that the participants identified as significant [27]. Key questions asked participants to describe: noteworthy successes and challenges faced during GHE participation; resources they required (both those present and those missing in the setting); and the perceived impact of GHEs on themselves, on the host country, or on other affected individuals or structures (Additional files 1 and 2). On average, interviews lasted 60 minutes. A qualitatively trained research assistant conducted the interview in a conversational style. Probes and reflective statements encouraged participants to provide additional details and sought clarification. Interviews were digitally recorded and rendered anonymous during transcription. Two transcriptionists reviewed the transcripts to ensure descriptive validity [28].

### Data analysis

As per Sandelowski’s recommendation [24], we used content analysis as our analysis strategy. More specifically, we employed conventional content analysis [29] to generate coding themes. As interviews were conducted, each research team member reviewed transcripts independently to identify and define emergent themes. Regular team meetings were held to discuss these themes in order to develop a consensus-derived thematic structure. Using the constant comparison technique [26], additional interviews were held to vet the coding structure until theoretical saturation [27] was achieved. This iterative process provided methodological rigor as initial themes were revised and refined through subsequent data collection and analysis. Coded data was then grouped and organized using NVivo software to facilitate cross-referencing (Version 7; QSR International Pty Ltd, Doncaster, Vic, Australia).

This paper presents the findings and resulting conceptualization developed during the collection and analysis of the entire dataset. It should be noted that a single thematic structure sub-node related to medical tourism has been previously published [10]. In contrast to the previous publication, this paper is a broad-scope analysis of the complex relationships and expectations that arise during GHEs.

## Results

Interviewees identified three groups of interested parties involved in their GHEs: participants, host communities, and sponsoring institutions. Around these groups, four major themes emerged: (i) cultural challenges, (ii) expectations and perceptions, (iii) relationships and communication, and (iv) discordant objectives. In the context of each theme, participants frequently described tensions existing between the stakeholder groups. To illustrate these results, we selected interview excerpts that represent each theme. Participants are identified by an interview number and their status as a trainee (T) or professional (P).

### Cultural challenges

Participants reported cultural differences as a key inherent challenge in GHEs. They repeatedly described the challenges of accepting the cultural practices of host communities while preserving their own moral and cultural ideals. For example, one medical student described the challenge to:

“find a place between respecting other people’s cultures and not imposing my own views even when some of the cultural activities were partly responsible for spreading certain diseases.” (19 T)

Another medical student described cultural tensions in the context of treatment:

“Antiretrovirals were seen as making the patients sick…so they would throw them out…they were coming from the culture where anything that a white person gives you is to be mistrusted to a certain degree.” (14 T)

An experienced healthcare professional discussed cultural differences as the greatest challenge during GHEs, citing the discrepancy in perceptions of health and wellness based on culture:

“You go there with your own cultural biases and you go there with your own work ethic and you go there with your own vision of what health is and what medicine is and what health care is… [global health participants] are often frustrated and become judgmental.” (4 T)

Some participants acknowledged that greater experience abroad diminished frustrations associated with cultural differences during healthcare delivery.

### Expectations and perceptions

In addition to cultural challenges, participants struggled to meet the expectations of host communities. All trainee participants stated that their main goal during their GHEs was to experience healthcare in low-resource settings. Notwithstanding this expressed goal, most trainees felt uncertain about their clinical roles in the GHE setting. Trainees acknowledged that their level of clinical training at the time of the GHE limited their contributions. Several expressed feelings of failure because their clinical skills did not meet the expectations of host communities. One pre-clinical medical student described such a feeling after she declined to perform procedures beyond her clinical skill set:

“It was definitely embarrassing and you got a lot of laughs, like: “What do you mean you came all the way here and you don’t know how to do those things. So what did you come here for?” ” (7 T)

Another trainee discussed the challenges of unrealistic expectations from the host community and how that can contribute to feeling uncomfortable in a clinical setting:

“There is an expectation when you’re coming from a developed country that your knowledge base is a lot high than it necessarily is and…it is a bit disconcerting because you are actually going to a place when you have very little experience.” (20 T)

The optimal timing for overseas participation was also mentioned in relation to trainee expectations. Some trainees questioned whether pre-clinical participation in GHEs was appropriate given the lack of medical knowledge especially when host community expectations could not be met. One trainee remarked:

“I don’t think I should have gone after 1^st^ year medical school. I think you definitely need to be at least 3^rd^ year (clinical year) because you are too dependent on being taught…You can’t take a patient load on your own” (1 T)

Clinical supervisors from host communities were often unfamiliar with the levels of trainee experience. Trainee objectives were either lacking or different from the expectations of the host community. The professional participants echoed these sentiments, describing watching trainees flounder during GHEs. Defined objectives, personal and professional safety nets and well-established electives were all important factors in successful GHEs. One experienced professional in global health emphasized the benefit of more structured GHEs for those beginning their global health careers:

“you have to get involved with an organization that is very well structured and the more structured the organization, the more it protects you as an individual.” (17P)

While trainees experienced tensions with host communities regarding clinical skill expectations, professionals described tensions with hosts in overall project design. The two groups often had conflicting goals and objectives, as summarized by one professional:

“I think the thing that I found hardest is dealing with local surgeons to develop an educational project. It is not that they are not willing, but they have their own barriers to making something happen. Some of it is lack of resources, some of it is cultural differences and some of it is, I suspect, our unconscious projection of expectations which perhaps are beyond what they want.” (2P)

### Relationships & communication

Both trainee and professional participants agreed that developing and/or maintaining relationships with host communities was challenging. Participants described being labeled with a pejorative “Western superiority” that they either personally experienced or observed in others when interacting with their host communities. One professional discussed this concept as it applied to most global health participants who travelled to low-resource settings:

“We give ourselves a certain air of superiority when we are there as donors, as helpers, as providers. It is a very unequal sort of relationship with the partners that we have there and, if you are serious about partnering and if you are serious about building capacity, teaching and helping, it is not the healthiest type of relationship.” (4P)

Generally, this notion of “Western superiority” was discussed more by experienced professionals than by trainees. Professionals stressed that respect and communication were key components to improving relationships with host communities. One experienced professional summarized these ideas stating:

“We forget that the people work with limited resources and they do the best they can with what they have got and they are working with the knowledge that they have. And that doesn’t make us better. It doesn’t make them worse. It is just the situation as it is and I think that we often forget that people have a lot to teach us.” (4P)

Several trainees noted the importance of trust as a key element in building relations with host communities. One trainee discussed the challenge of establishing trust with both local physicians and patients:

“you never knew whether they [patients] trusted us enough or how much trust there is in that relationship, which is really important obviously.” (14 T)

The challenges in relationship-building were not exclusive to the participant-host relationship. Similar issues arose between hosts and sponsoring institutions. One participant recalled a failing relationship between a host community and a sponsoring institution, resulting in an unsuccessful project:

“I was in a country where [a donor] had built a beautiful hospital…and there wasn’t one person or patient in it. They were still using the old hospital. Nobody asked them what the hospital should look like…[the hospital] was built according to our model but culturally it didn’t work for them.” (4P)

### Discordant objectives

Beyond relationships and communication, participants described facing challenges of conflicting objectives between themselves and sponsoring institutions. Many professional participants described projects that were completed without support from local communities, resulting in underutilized facilities or services. Professionals were acutely aware of the need to ensure that their own goals were aligned with those of both the host communities and the sponsoring institutions. For example, one participant described necessary criteria for future projects:

“finding a worthwhile project that both meets my learning objectives and professional objectives to create something that is sustainable in the host country or the host project and also giving to the country.” (6P)

In several instances, professional participants encouraged trainees to seek well-established projects early in their global health career. Such projects are likely to have objectives approved by all stakeholders involved as outlined by one professional participant:

“The more structure the organization, the less unexpected events there will be. You go there [to the host community] and the team has been there for the past ten years. The locals know you. The team knows the locals and the project is very well defined. Those are the types of projects you want to get involved in when you are starting off.” (17P)

In contrast, misaligned objectives can be a significant challenge during GHEs as described by one healthcare professional:

“all the expert reports I read, everybody I talked to in NGO’s, governments here, governments there, they said: “what people will want is antiretroviral drugs.” When I did extensive community consultation, it came 5^th^ on the list. Way before drugs, they wanted food, then they wanted education for their children.” (12P)

Participants frequently reported tensions and conflicting objectives with the organization or agency involved in sponsoring the GHE. Trainees felt that having better defined objectives from the sponsoring institution would have improved the overall experience. As expressed by one participant:

“it would have been nice to sit down with someone, a mentor, someone to discuss what were the personal goals, what were our objectives for going and what you planned as a medical student to do there.” (14 T)

## Discussion

The four themes emerging from our analysis involved three GHE stakeholder groups: participants, hosts and sponsoring institutions. Given that interviewees were all GHE participants, much of the discussion focused on tensions between participant-host and participant-sponsor groups. Cultural issues were identified as one of the primary sources of tension from the participants’ perspectives as they continually balanced their own beliefs with those of host communities. This highlights the need for participants to engage in adequate pre-departure training that includes focus on cultural specificities of host communities. Participants, particularly trainees, felt inadequate and uncomfortable when their clinical skills did not meet their hosts’ expectations, which was a regular occurrence. This requires participants to inform hosts of their personal GHE expectations and abilities; however, some responsibility must also lie with both sending and receiving training institutions to facilitate proper learning objectives and host expectations.

Among the non-participant related tensions, most related to conflicting project objectives between hosts and project sponsors. This occurred when sponsoring institutions did not adequately consider the needs of host communities, such as sending antiretroviral drugs when nutrition was a greater concern to community members. Interestingly, only experienced participants made such observations. This may indicate that experience within project decision-making is required to observe such interactions, positions unlikely to be undertaken by trainees. Despite this lack of awareness, these tensions must be conveyed to trainees so that they may be adequately prepared in the event that they encounter such conflicts. Furthermore, such knowledge will help trainees better understand the complexities of global health projects and the potential struggles that exist during project development and deployment.

Pragmatically, trainees should be encouraged to participate in GHEs that are well-vetted by their sending institutions. Such vetting would ensure that the host community understands the limitations of the trainees, and would seek to minimize the conflicting objectives among stakeholders. Furthermore, pre-departure training would inform prospective participants about the perspectives and expectations of host communities. While this study does not enable or describe such host perspectives, other research does provide insights into these important considerations. One recent example of such research describes the perspectives of Malawian medical trainees regarding international medical trainees who travel to their hospital for “clinical tourism” [30]. The quotes and analyses in this publication offer important viewpoints that should be considered by trainees who will participate in these overseas experiences.

GHEs are relatively new training opportunities within modern medical education programs. While there are numerous advantages to GHEs, it is essential to prepare trainees to contend with tensions that may arise between GHE groups. This study enables us to better understand and conceptualize these tensions. We suggest that three stakeholder groups are involved in GHEs: GHE participants, host communities, and sponsoring bodies. Equal importance should be attributed to each group because appropriate consideration to the needs and objectives of each stakeholder is essential to the attainment of GHE success [31]. While each stakeholder group will have their own objectives, success will be maximized when these objectives are shared or are maximally congruent across groups. To illustrate, consider the following example of a medical student on a surgical ward of a resource-poor setting where the primary objective for each stakeholder confers mutual benefit between groups (Table 1).

**Table 1:** Example of congruent objectives experienced in a successful GHE

**Stakeholder group**	**Objective**
Individual GHE participant	Experience health care delivery in resource-poor setting in the operating room and hospital surgery ward.
Host community	Receive participant who provides health care support commensurate with skill.
Sponsoring body	Offer the participant a well-supervised clinical experience in a resource-poor setting that supports the needs of the host community.

**Figure 1:**
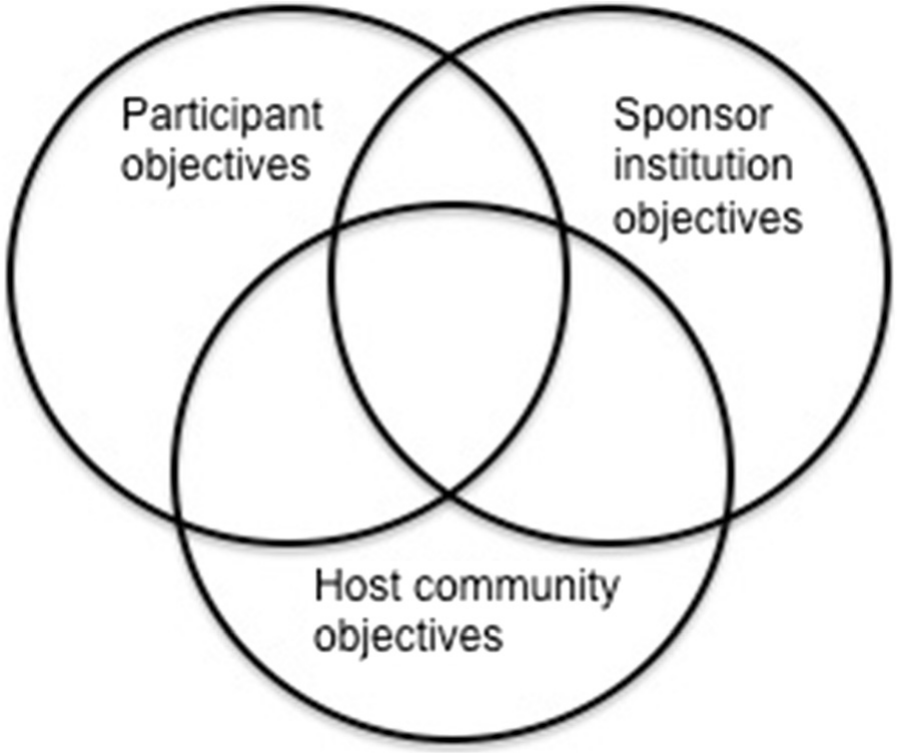
Depiction of how shared objectives can benefit each stakeholder.

This ideal sharing of objectives is depicted in Figure 1, where common objectives are represented by the overlapping of circles in the Venn diagram.

We propose that this conception of shared objectives should inform the development of global health curricula, and especially the formulation of global health educational objectives for trainees.

**Figure 2:**
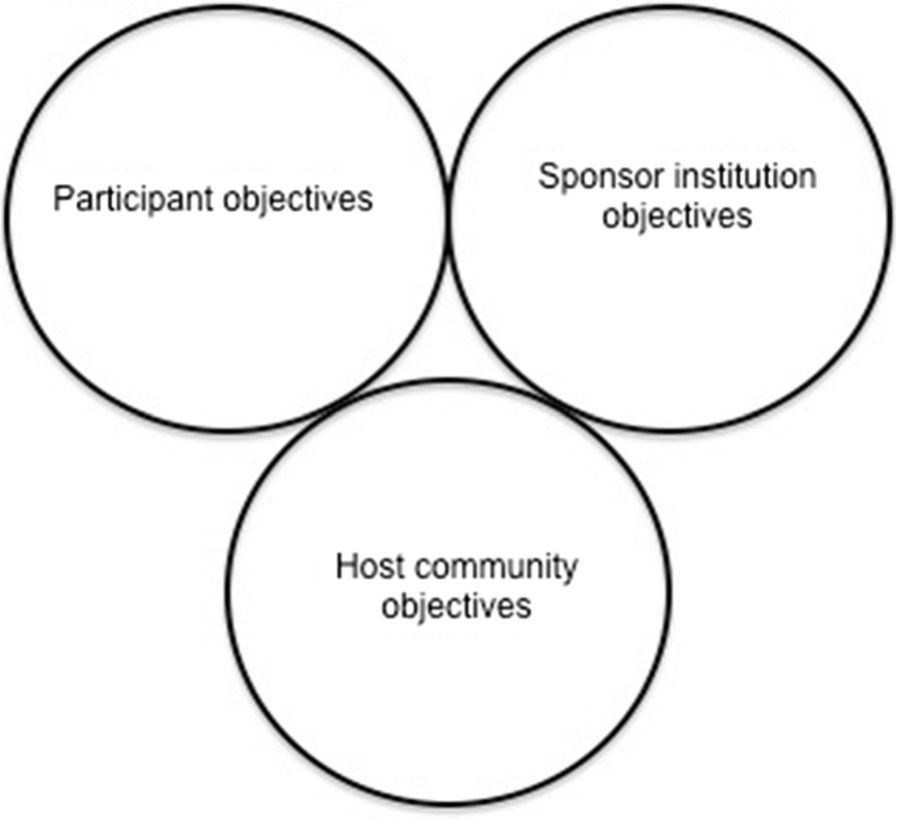
Depiction on how incompatible objectives can jeopardize the success of a GHE for each stakeholder.

Perhaps even more important to the success of GHEs is the reduction of objectives that confer no benefit or that may harm other stakeholders. To illustrate, consider this example of a medical student who is asked to undertake significant responsibility during a GHE (Table 2). The primary objectives for each stakeholder are incommensurable with those of the other two groups. This lack of congruent objectives is depicted in Figure 2, where the objectives of each stakeholder group are represented by circles that do not overlap.

**Table 2:** Example of incompatible objectives experienced in an unsuccessful GHE

**Stakeholder group**	**Objective**
Individual GHE participant	Gain trainee-level, supervised surgical experience in resource-poor setting.
Host community	Accept trainee with expectation that they will work independently and relieve local surgeons.
Sponsoring body	Increase surgical capacity in host community with instruction of local surgical trainees by visiting trainee participant.

We propose that these schemas represent useful conceptualizations for educators in the design of GHEs. We hope that they encourage dialogue between stakeholder groups to ensure mutual and maximal benefit.

Global health activities continue to gain momentum in medical education as increasing numbers of medical trainees (both post-graduate and undergraduate) participate in GHEs [32,33]. While research is investigating potential benefits and harms to trainees, little is known about the relationships and interactions between trainees and other GHE stakeholders. A recent guideline for ethics and best practice in global health training was developed with particular attention to several GHE stakeholders [31]. The authors of this guideline acknowledge the benefits of GHEs but also the potential ethical challenges and burdens on hosts. These guidelines were developed based on available literature and author experience; however, the lack of evidence to guide several recommendations was noted. Our study provides additional evidence to support their recommendations, particularly the need for established, congruent objectives and the importance of communication between all stakeholders.

We acknowledge several limitations to this study. Most notably, we have no data collected from host communities or sponsoring bodies. As others have experienced when investigating educationally-oriented GHEs, daunting obstacles impede the collection of data from these groups. With respect to host communities, language barriers, perceived power differentials, and logistics such as time zone differences and travel costs are just some of the barriers that curtail ready access to host community participants. Regarding sponsoring bodies, securing the participation of well-placed and well-informed insiders, and negotiating sponsor alliances to religious or other institutions are some hurdles that must be overcome. While these obstacles are not insurmountable, it was not within the scope of this needs assessment study’s mandate, or budget, to invest the time required to conquer them. In the tradition of Glaser and Strauss [34], we present this conceptualization as an ever-developing entity – one that will evolve as the data-“grounded” findings from other studies continue its vetting. Given the obstacles hindering in-country and sponsor-related data, we hope that the works of others can contribute to the evolution of this “momentary product” [34].

This study did sample both medical trainees and health professionals with a broad range of GHEs and perspectives. Indeed, some participants had GHEs spanning several decades. Although this research was carried out in a single Canadian university, the results are more representative given our participants had 103 experiences in 21 countries. Furthermore, the recurrent themes we identified in the study, across the heterogeneity of our study population suggest that the stakeholder tensions described here are pervasive across a range of GHEs. We present these themes for further investigation by global health and medical education researchers when developing electives and curricula. Indeed, as the results from this study indicate, the perspectives and experiences of host communities and sponsoring agencies must be included in future studies if medical schools are to responsibly prepare trainees for GHEs.

## Conclusion

This study demonstrates that global health participants frequently face substantial tensions with other global health stakeholders during GHEs. Trainees are particularly vulnerable as they lack the experience and knowledge to navigate these tensions. Of considerable concern is the mismatch of expectations between each group and the potential for conflicting objectives. However, GHEs must not be abandoned as considerable evidence exists regarding their potential benefits. Instead, a balance should be sought that acknowledges the mutually-compatible needs of each group. Our findings highlight the importance of trainees participating in GHEs with well-designed objectives that maximize benefit for all stakeholders. We propose a conceptualization for the development of educational objectives that acknowledges participants, host communities and sponsoring institutions. Aligning each group’s objectives will improve the experiences for all.

In conclusion, we feel our descriptions, suggested conceptualizations, and recommendations provide data-grounded analysis that can meaningfully and usefully contribute to the effort to critically analyze and to appropriately prepare our trainees to participate in GHEs.

## Abbreviations

GHE: Global health experiences; T: Trainee; P: Professional; NGO: Non-governmental organization.

## Competing interests

The authors declare that they have no competing interests.

## Authors’ contributions

AM conceived the study concept and design, analyzed and interpreted data, provided critical revisions to the manuscript and organized administrative and material support for the study. AP collected, analyzed and interpreted the data, drafted the manuscript and provided critical revisions to the manuscript. LV conceived the study concept and design, provided extensive expertise regarding the study methodology and provided critical revisions to the manuscript. All authors read and approved the final manuscript.

## Authors’ information

AM is a staff physician and Professor of Medicine and Director of the Office of Global Health with the Faculty of Medicine, University of Ottawa. She has twenty years global health experience, participating and preparing health care providers. Her medical education research centers around development of educational resources for those undertaking global health experiences.

AP is an emergency medicine resident at the University of Toronto and currently enrolled in the Masters of Medical Education Leadership program at the University of New England. His recent work includes procedural skill acquisition among trainees and global health curriculum design.

LV is Assistant Professor at the Academy for Innovation in Medical Education (AIME) at the University of Ottawa. She is a qualitative researcher (PhD) with interests in Activity Theory, Actor Network Theory and Chaos. Her recent work applies these theories to interprofessional collaborations / education and electronic patient records.

## Pre-publication history

The pre-publication history for this paper can be accessed here:

http://www.biomedcentral.com/1472-6920/13/136/prepub

## Supplementary Material

Additional file 1 Survey – Global Health Initiative – Professionals form. Click here for file

Additional file 2 Survey – Global Health Initiative – Student form. Click here for file

## References

[B1] Brewer TF Saba N Clair V From boutique to basic: a call for standardised medical education in global health Med Educ 2009 43 10 930 933 19769640 10.1111/j.1365-2923.2009.03458.x

[B2] Drain PK Primack A Hunt DD Fawzi WW Holmes KK Gardner P Global health in medical education: a call for more training and opportunities Acad Med 2007 82 3 226 230 17327707 10.1097/ACM.0b013e3180305cf9

[B3] Eckhert NL Getting the most out of medical students' global health experiences Ann Fam Med 2006 4 Suppl 1 S38 39 discussion S58-60 17003161 10.1370/afm.563 PMC1578668

[B4] Epstein RM Assessment in medical education N Engl J Med 2007 356 4 387 396 17251535 10.1056/NEJMra054784

[B5] Houpt ER Pearson RD Hall TL Three domains of competency in global health education: recommendations for all medical students Acad Med 2007 82 3 222 225 17327706 10.1097/ACM.0b013e3180305c10

[B6] Ramsey AH Haq C Gjerde CL Rothenberg D Career influence of an international health experience during medical school Fam Med 2004 36 6 412 416 15181553

[B7] Banatvala N Doyal L Knowing when to say "no" on the student elective. Students going on electives abroad need clinical guidelines BMJ 1998 316 7142 1404 1405 9572746 10.1136/bmj.316.7142.1404 PMC1113113

[B8] Edwards R Piachaud J Rowson M Miranda J Understanding global health issues: are international medical electives the answer? Med Educ 2004 38 7 688 690 15200392 10.1111/j.1365-2929.2004.01849.x

[B9] Thompson MJ Huntington MK Hunt DD Pinsky LE Brodie JJ Educational effects of international health electives on U.S. and Canadian medical students and residents: a literature review Acad Med 2003 78 3 342 347 12634222 10.1097/00001888-200303000-00023

[B10] Petrosoniak A McCarthy A Varpio L International health electives: thematic results of student and professional interviews Med Educ 2010 44 7 683 689 20636587 10.1111/j.1365-2923.2010.03688.x

[B11] Elit L Hunt M Redwood-Campbell L Ranford J Adelson N Schwartz L Ethical issues encountered by medical students during international health electives Med Educ 2011 45 7 704 711 21649703 10.1111/j.1365-2923.2011.03936.x

[B12] Sharafeldin E Soonawala D Vandenbroucke JP Hack E Visser LG Health risks encountered by Dutch medical students during an elective in the tropics and the quality and comprehensiveness of pre-and post-travel care BMC Med Educ 2010 10 89 21126347 10.1186/1472-6920-10-89 PMC3014955

[B13] Dowell J Merrylees N Electives: isn't it time for a change? Med Educ 2009 43 2 121 126 19161481 10.1111/j.1365-2923.2008.03253.x

[B14] Shah S Wu T The medical student global health experience: professionalism and ethical implications J Med Ethics 2008 34 5 375 378 18448720 10.1136/jme.2006.019265

[B15] Bozorgmehr K Schubert K Menzel-Severing J Tinnemann P Global health education: a cross-sectional study among German medical students to identify needs, deficits and potential benefits (part 1 of 2: mobility patterns & educational needs and demands) BMC Med Educ 2010 10 66 20932277 10.1186/1472-6920-10-66 PMC2958967

[B16] Haq C Rothenberg D Gjerde C Bobula J Wilson C Bickley L Cardelle A Joseph A New world views: preparing physicians in training for global health work Fam Med 2000 32 8 566 572 11002868

[B17] Drain PK Holmes KK Skeff KM Hall TL Gardner P Global health training and international clinical rotations during residency: current status, needs, and opportunities Acad Med 2009 84 3 320 325 19240438 10.1097/ACM.0b013e3181970a37 PMC3998377

[B18] Developing global health curricula: A guidebook for US medical schools [http://globalhealthedu.org/PublicDocs/Developing%20GH%20Curricula_Guidebook%20for%20US%20Medical%20Schools.pdf]

[B19] Battat R Seidman G Chadi N Chanda MY Nehme J Hulme J Li A Faridi N Brewer TF Global health competencies and approaches in medical education: a literature review BMC Med Educ 2010 10 94 21176226 10.1186/1472-6920-10-94 PMC3019190

[B20] McKinley DW Williams SR Norcini JJ Anderson MB International exchange programs and U.S. medical schools Acad Med 2008 83 10 Suppl S53 57 18820502 10.1097/ACM.0b013e318183e351

[B21] Grudzen CR Legome E Loss of international medical experiences: knowledge, attitudes and skills at risk BMC Med Educ 2007 7 47 18045481 10.1186/1472-6920-7-47 PMC2242732

[B22] Suchdev PS Shah A Derby KS Hall L Schubert C Pak-Gorstein S Howard C Wagner S Anspacher M Staton D A proposed model curriculum in global child health for pediatric residents Acad Pediatr 2012 12 3 229 237 22484282 10.1016/j.acap.2012.02.003

[B23] Riner ME Globally engaged nursing education: an academic program framework Nurs Outlook 2011 59 6 308 317 21784495 10.1016/j.outlook.2011.04.005

[B24] Sandelowski M Whatever happened to qualitative description? Res Nurs Health 2000 23 4 334 340 10940958 10.1002/1098-240x(200008)23:4<334::aid-nur9>3.0.co;2-g

[B25] Kuzel A Crabtree B, Miller W Sampling in qualitative inquiry Doing qualitative research 1999 Thousand Oaks: Sage Publications 33 45

[B26] Charmaz K Denzin N, Lincoln Y Grounded theory: objectivist and constructivist methods Handbook of qualitative research 2000 2 Thousand Oaks, CA: Sage Publications, Inc

[B27] Charmaz K Constructing Grounded Theory: A practical guide through qualitative analysis 2006 Thousand Oaks, California: Sage Publications

[B28] Maxwell JA Understanding and validity in qualitative research Harv Educ Rev 1992 62 3 279 300

[B29] Hsieh HF Shannon SE Three approaches to qualitative content analysis Qual Health Res 2005 15 9 1277 1288 16204405 10.1177/1049732305276687

[B30] Wendland CL Moral maps and medical imaginaries: clinical tourism at Malawi's college of medicine Am Anthropol 2012 114 1 108 122 22662357 10.1111/j.1548-1433.2011.01400.x

[B31] Crump JA Sugarman J Ethics and best practice guidelines for training experiences in global health Am J Trop Med Hyg 2010 83 6 1178 1182 21118918 10.4269/ajtmh.2010.10-0527 PMC2990028

[B32] Anderson KC Slatnik MA Pereira I Cheung E Xu K Brewer TF Are we there yet? Preparing Canadian medical students for global health electives Acad Med 2012 87 2 206 209 22189881 10.1097/ACM.0b013e31823e23d4

[B33] Howard CR Gladding SP Kiguli S Andrews JS John CC Development of a competency-based curriculum in global child health Acad Med 2011 86 4 521 528 21346499 10.1097/ACM.0b013e31820df4c1

[B34] Glaser B Strauss A The Discovery of Grounded Theory: Strategies for qualitative research 1967 Piscataway, NJ: Transaction Publishers

