# Supplementary material for: The complex relationships involved in global health: a qualitative description
Source: BMC Med Educ. 2013 Oct 3;13:136. doi: 10.1186/1472-6920-13-136 (PMC3819699; doi:10.1186/1472-6920-13-136)
Supplement: Additional file 2 — Survey – Global Health Initiative – Student form. [file 1472-6920-13-136-S2.pdf]

Survey – Global Health Initiative – Student form

Thank you for agreeing to participate in the Global Health Initiative survey. The survey will consist of two sections.

Part One is a demographic and global health profile questionnaire that will attempt to capture your involvement in global health activities. By participating in this part of the interview, you allowing us to collect and post this information on-line, and to potentially act as a mentor in the global health community. With your permission, your answers will be used to construct your profile on our website. Consequently, none of the information obtained from this section will be anonymous.

Part Two is a needs' assessment in which we will focus on several key themes of global health and ask you to describe your own experiences. Your responses will be used to inform the development of a global health curriculum. In this section, I will be audio recording your answers. This part of the interview is a conversation-style answer and response. So at times, I may at ask you to further expand on your experiences. Please note, that this portion of the survey will be entirely anonymous, and remain separate from part one, above. The anonymous audio-recordings will be transcribed and only the transcriptions will be used for data analysis.

If you require any clarification of a question(s), please stop and ask prior to responding and I will better explain the question.

We will now begin.

CONTACT INFORMATION

1. Name:

2. Email:

3. Telephone (work):

What is your preferred method of contact – email or phone

PART ONE: PARTICIPANT'S DEMOGRAPHIC & GLOBAL HEALTH PROFILE

4. Education (please specify your *degree(s)* and *name(s) of institution(s)* where they were obtained):
   - MD: _______ Institution: __________________
   - RN: _______ Institution: __________________
   - MSc: ______ Institution: __________________
   - PhD: ______ Institution: __________________
   - Specialty &/or Subspecialty: _________ Institution: __________________
   - Other – please specify: ____

5. Can you specify your current program and year:
   - **Medical** *pre-clinical (yr 1-2)*: ______ *clinical* (yr 3-4): ________

- **Nursing**: _____
- **Master's**: _____
- **Resident** (specify specialty & year): _________________
- **Undergraduate Health sciences**: ____
- **Other** (please specify): ________________________

6. Are you affiliated with an NGO? YES NO
   If **YES**, please specify:

7. Do you have any affiliations with a group(s) related to Global/International Health – (*including student organized or professional*)
   YES NO
   If **YES**, please specify:

8. Area(s) of interest related to Global Health (please specify all that apply):
   - basic science/laboratory: ____
   - epidemiology: ____
   - clinical practice: ____
   - medical education: ____
   - health policy: ____
   - other: (please specify) _____________________________

9. Have you ever lived, volunteered or worked in a resource poor country? (If YES please proceed to question 10, if NO, please proceed to question 11)
   YES NO

10. Please list countries and check the appropriate categories that best identify the nature of your work.

**COUNTRY:** ____________ **YEAR:** ________ **DURATION:** _______________	
**CLINICAL AREA(S)**	**NON-CLINICAL AREA(S)**
________________________________<br>________________________________<br>________________________________<br>________________________________	________________________________<br>________________________________<br>________________________________<br>________________________________
(If prompted to define clinical areas: **PROMPT** "*A clinical area is, for example,*")<br>HIV/AIDS: ___<br>Obstetrics/Gynecology: ___<br>Pediatrics: ___<br>Surgery: ___	(If prompted to define non-clinical areas: **PROMPT** *"A non-clinical area is, for example,"*)<br>Basic science/Laboratory research: ___<br>Health Policy: ___<br>Medical Education: ___<br>Project management: ___

COUNTRY: ___________ YEAR: _______ DURATION: ______________	
**CLINICAL AREA(S)**	**NON-CLINICAL AREA(S)**
____________________<br>____________________<br>____________________<br>____________________<br><br>(If prompted to define clinical areas: **PROMPT** "*A clinical area is, for example*,")<br>HIV/AIDS: ___<br>Obstetrics/Gynecology: ___<br>Pediatrics: ___<br>Surgery: ___	____________________<br>____________________<br>____________________<br>____________________<br><br>(If prompted to define non-clinical areas: **PROMPT** *"A non-clinical area is, for example,"*)<br>Basic science/Laboratory research: ___<br>Health Policy: ___<br>Medical Education: ___<br>Project management: ___

COUNTRY: ___________ YEAR: _______ DURATION: ______________	
**CLINICAL AREA(S)**	**NON-CLINICAL AREA(S)**
____________________<br>____________________<br>____________________<br>____________________<br><br>(If prompted to define clinical areas: **PROMPT** "*A clinical area is, for example*,")<br>HIV/AIDS: ___<br>Obstetrics/Gynecology: ___<br>Pediatrics: ___<br>Surgery: ___	____________________<br>____________________<br>____________________<br>____________________<br><br>(If prompted to define non-clinical areas: **PROMPT** *"A non-clinical area is, for example,"*)<br>Basic science/Laboratory research: ___<br>Health Policy: ___<br>Medical Education: ___<br>Project management: ___

COUNTRY: ___________ YEAR: _______ DURATION: ______________	
**CLINICAL AREA(S)**	**NON-CLINICAL AREA(S)**
____________________<br>____________________	____________________<br>____________________

___	___
(If prompted to define clinical areas: **PROMPT** "*A clinical area is, for example*,")<br>HIV/AIDS: ___<br>Obstetrics/Gynecology: ___<br>Pediatrics: ___<br>Surgery: ___	(If prompted to define non-clinical areas: **PROMPT** *"A non-clinical area is, for example,"*)<br>Basic science/Laboratory research: ___<br>Health Policy: ___<br>Medical Education: ___<br>Project management: ___

11. Are you currently involved in any global health activities? (If YES please specify, If NO then proceed to question 12 if NO)

YES NO

___

___

___

___

12. Are you interested in participating in any future global health activities?

YES NO

If YES, please circle below to indicate in what capacity and duration of availability:

STUDENTS (please specify)

Pre-clinical	Local	Global	Duration:
Clinical	Local	Global	Duration:
POSTGRAD	Local	Global	Duration:
FACULTY	Local	Global	Duration:

(If applicable, please specify preference of specialty)

13. Are you willing to be a resource for global health activity in any of the following capacities?

    a. PEER Mentor

    b. Educational presentations/teaching - specific topic(s) of interest:

    c. Other (please specify): ____

PART TWO: NEEDS ASSESSMENT

We will now commence the needs' assessment portion of the survey. I remind you that this section is entirely anonymous and your responses will be audio-recorded and transcribed.
I encourage you throughout this section to please describe in your own words, the experiences that you think are most relevant. Do you have any questions before we begin?

1. Please describe the most significant challenges/barriers you have encountered in your Global Health work.

    a) Related to the example(s) above, can you please describe how these issues were coped with or resolved?

    b) Are there any other significant challenges that you would like to discuss that we have not covered?

2. Please describe some noteworthy successes you experienced during your global health work that were not included in question #1.

3. Please describe any resources that you found most beneficial during your Global health project(s). This may be prior to your departure, when you were abroad and/or when you returned home. (If asked for clarification of the term "resources"; PROMPT: "*for example, resources might include courses, funding, contacts, debriefing opportunities*")
    a) Follow up question: Can you think of resources that you wish you had had available?

4. Why do you go abroad, and what drives you to continue your work?

5. Have your experiences impacted your practice and/or life? Please expand.

6. Are there any topics or themes that you believe to be essential in the field of global health such that they should be taught to all medical students regardless of whether they will have future involvement in the field? If so, please specify. These topics or themes may be presented in the context of PBLs, a mandatory course or general inclusion throughout the curriculum.

7. Another topic we're interested in exploring is your experiences of interprofessional collaboration in your Global Health activities. And we understand that the idea of 'interprofessional' may be very different for in your global health work than in your Canadian practices. Could comment on your experiences of collaborating with other healthcare professionals in global health settings? Maybe compare these to your experiences here in Canada?

8. Please tell me about the perceptions of the impacts your GH work had on individuals and/or communities in the host country. These perceptions can be your own or those from people of the host country.

b) What does medical tourism mean to you? Have you encountered it?

9. We have had a chance to talk about some of the aspects of your global health activities. There may be issues that haven't been addressed yet, can you describe to me any other issues and themes that you would like to address.

10. Please describe any advantages or disadvantages that you could foresee in establishing a network of information sharing & resource pooling amongst Global Health contributors through the Global Health Initiative?

11. During your global health experiences, were there any occasions in which you or other trainees were involved in situations where you were concerned about patient safety?
    a) Can you give me an example or a story about such an experience?

12. While you were abroad, were there any clinical situations where you were concerned about your own or another trainee's personal safety?
    a) Can you describe that situation for me?

13. During your global health experiences, were there any instances where you or another trainees were asked or felt pressured to perform above your clinical competency or skill level?
    a) Can you give an example or a story?

14. While you were abroad, were there any clinical situations that you would describe as ethically questionable either involving you or other trainees?

    a) Can you tell me about that situation?

This concludes the needs' assessment portion of our survey. I would like to thank you very much for taking the time to aid us in building a stronger global health community. Your time is greatly appreciated.
